# The Potential of Plant Growth-Promoting Fungi Enhances the Growth, Yield, and Phytochemical Compounds of *Oryza sativa* L. (Maled Phai Cultivar) Under Field Conditions

**DOI:** 10.3390/plants14121839

**Published:** 2025-06-15

**Authors:** Wasan Seemakram, Sabaiporn Nacoon, Jindarat Ekprasert, Piyada Theerakulpisut, Jirawat Sanitchon, Sophon Boonlue

**Affiliations:** 1 Department of Microbiology and Parasitology, Faculty of Medical Science, Naresuan University, Phitsanulok 65000, Thailand; seemakram.w@gmail.com; 2 Department of Microbiology, Faculty of Science, Khon Kaen University, Khon Kaen 40002, Thailand; ning502@gmail.com (S.N.); jindaek@kku.ac.th (J.E.); 3 Department of Biology, Faculty of Science, Khon Kaen University, Khon Kaen 40002, Thailand; piythe@kku.ac.th; 4 Salt-Tolerant Rice Research Group, Khon Kaen University, Khon Kaen 40002, Thailand; 5 Department of Agronomy, Faculty of Agriculture, Khon Kaen University, Khon Kaen 40002, Thailand; jirawat@kku.ac.th

**Keywords:** Maled Phai rice, organic agriculture, plant growth promoter, rhizosphere, upland rice

## Abstract

Excessive application of a chemical fertilizer during rice cultivation leads to soil infertility and increases production costs. An alternative way to reduce over-fertilization is to partially or fully replace the fertilizer with microbes that promote the growth and production of plants. This study aimed to investigate the Maled Phai rice cultivar (*Oryza sativa* L.) in a field experiment with two fungi strains. *Rhizophagus variabilis* KS-02 and *Trichoderma zelobreve* PBMP16 were selected as inocula and compared with non-*R. variabilis* KS-02 and non-*T. zelobreve* PBMP16, acting as controls, one without synthetic fertilizer and one with synthetic NPK fertilizer. The field experiment was conducted in a Randomized Complete Block design with four replications. Growth and yield parameters were determined in the plant tissues and roots, and bioactive compounds were determined in the rice seeds. The results show the presence of *T. zelobreve* PBMP16 and *R. variabilis* KS-02 colonization in the plant roots at the harvest stage. A single inoculum of both *R. variabilis* KS-02 and *T. zelobreve* PBMP16 significantly increased most of the plant growth performance and yield parameters, as well as the concentrations of bioactive compounds. Remarkably, such effects were more apparent than those observed with the use of a chemical fertilizer. Thus, a single inoculum of *R. variabilis* KS-02 or *T. zelobreve* PBMP16 and the co-inoculation of both have the potential to increase the grain yield and bioactive compounds of Maled Phai under field conditions.

## 1. Introduction

Rice is one of the most important staple food crops in several countries, consumed by over half of the world’s population, with approximately 95% of production occurring in Asia [1]. Many special cultivars of rice contain color pigments, such as black rice, red rice, and brown rice. Among them, Maled Phai, a cultivar of black rice, is widely cultivated in Thailand. The Maled Phai rice cultivar’s nutritional values are as follows: 3.6–3.7% fat, 8.0–11.0% protein, 3.0–4.0% total dietary fiber, 70–75% total carbohydrate, and 360–365% calories [2]. It contains high levels of bioactive compounds, specifically anthocyanin and phenolic compounds, which contribute to its antioxidant activity, making it beneficial as a natural food colorant and functional food ingredient [3,4]. Therefore, increasing rice crop production by integrating nutrient management strategies, such as through the application of biofertilizers, is considered the most effective method for maintaining a healthy and sustainable soil system while increasing crop productivity.

To obtain a high yield of cultivated rice, a large amount of chemical fertilizer is required, which is a hazard to human health and causes environmental pollution [5]. To make rice cultivation more sustainable and less dependent on chemical fertilizers, the use of plant growth-promoting microorganisms (PGPMs) has recently become of great interest to improve rice growth and production [6]. There are several organisms across fungi kingdoms that can serve as PGPMs, making them a viable option to replace chemical fertilizers.

Arbuscular mycorrhizal fungi (AMF), one of the most common plant growth-promoting fungi (PGPF) in the environment, is a group of fungi symbiotically residing with plant roots. The recent development of regulatory genes has helped to unravel the arbuscular mycorrhizal fungi interaction in rice to promote plant growth development and to enhance phosphorus use efficiency. Regulatory genes play a major role in improving crop establishment and survival and enhance soil quality by mobilizing mineral nutrients in exchange for carbon, resulting in increased crop resistance to various environmental stresses and pathogens [7]. Such effects have been reported across a wide variety of plants, including rice [8]. A previous study shows that AMF inoculation increases the yield and anthocyanin content of red rice [9]. Moreover, some AMF species have an induction effect on the production of secondary metabolites in all parts of plants [10]. Therefore, it is imperative to find suitable approaches to using AMF as a bioinoculant to improve plant production.

*Trichoderma*, as endophytic fungi, as biocontrol agents, and as PGPF, are beneficial fungi that colonize plant organs and can enhance plant growth through a wide range of mechanisms. Some of these mechanisms involve increasing access to macro- and micronutrients, while others involve the production of plant hormones or increasing the water acquisition rate [7]. *Trichoderma* is efficient in inducing plant growth through the production of plant hormones such as auxin, gibberellic acid (GA), cytokinins, abscisins, and ethylene [11]. These plant hormones, synthesized by *Trichoderma,* are known to serve as chemical mediators and signaling molecules for plant growth [12]. *Trichoderma* can also indirectly boost plant growth by assisting the plant in acquiring nutrients. In nutrient acquisition, through a process known as the ‘rhizophagy cycle’, *Trichoderma* can access nutrients in the soil and then transport them back to the plants, where they penetrate the root cells at the tips of the roots closest to the nutrient exudate zone [13]. Several studies have reported the potential of co-inoculating *Trichoderma* and AMF to promote plant growth, but their effects on the accumulation of secondary metabolites, such as phenolic compounds and anthocyanin, are still limited, especially under field conditions [14,15,16].

Our previous reports showed the efficiency of using the co-inoculants between AMF and *Trichoderma* to promote the growth and production of rice (Maled Phai cultivar) under greenhouse conditions [14,15]. Nevertheless, there is still a knowledge gap regarding the application of both AMF and *Trichoderma* in field experiments. Therefore, this study aims to investigate the combined effects of AMF and *Trichoderma* in promoting plant growth, yield, and phenolic compounds, as well as anthocyanin and antioxidant accumulation, in Maled Phai rice seeds under field conditions.

## 2. Results

### 2.1. Determination of Plant Growth and Harvesting

The growth and yield of Maled Phai at the harvesting stage in response to PGPF inoculation are shown in Table 1 (Figures S1–S4). The presence of PGPF significantly increased all plant growth parameters and the production of Maled Phai at the harvesting stage compared to uninoculated plants (T1). Interestingly, Maled Phai cultivation with *T. zelobreve* PBMP16 (T4) showed maximum growth and yield parameters, including height, the number of tillers, shoot dry weight, the number of panicles, and grain weight, with values of 100.65 cm, 19.18, 54.65 g, 12.00, and 4120.40 kg/ha, respectively, when compared to the control and plants undergoing chemical fertilizer treatment.

To investigate the effects of AMF, endophytic fungi, and their combination on the photosynthetic performance of Maled Phai, plant parameters relating to photosynthesis, i.e., the net photosynthesis rate (Pn), transpiration rate (Tr), stomatal conductance (gs), and water use efficiency (WUE) were measured in leaf samples, and the results are shown in Table 2. The results show that the contents of gs and Tr were not significantly different in all plants. Remarkably, the Pn, WUE, SPAD value, and total chlorophyll content in the treatments with a single inoculum of either AMF or *Trichoderma* (T3 and T4) and those that underwent co-inoculation (T5) were maximized compared to the uninoculated plants, and they were significantly better than those of the fertilizer-treated plants.

The root growth quality of Maled Phai at the harvesting stage, including factors such as root length, root surface area, root diameter, root volume, specific root length, and root tissue density, is shown in Table 3. The results indicate that a single inoculum of either AMF or *Trichoderma*, as well as co-inoculation with both PGPF, can significantly enhance the root dry weight, root length, and root surface area, obtaining better than plants undergoing treatment with chemical fertilizer (T2) and the control (T1). Plants inoculated with AMF (T3) showed the greatest root diameter and root volume compared to the plants treated with chemical fertilizer (T2) and the control (T1). Noticeably, chemical fertilizer could only increase the specific root length compared to the control treatment. These results emphasize that the application of either AMF or *Trichoderma* alone or co-inoculation with both was more beneficial to the root qualities of Maled Phai than the use of a chemical fertilizer.

The effects of different treatments on plant nutrient uptake are shown in Table 4. The results indicate that single inoculations (T3 and T4) and co-inoculation (T5) significantly increased the potassium and nitrogen concentrations compared to the control. Such effects were comparable to those of the chemical fertilizer. Moreover, phosphorus uptake was not significantly affected by any type of treatment. Thus, PGPF show promise as a replacement for chemical fertilizer to improve nutrient uptake.

### 2.2. Quantification of AMF Spores and Colonization in Plant Roots

**Figure 1:**
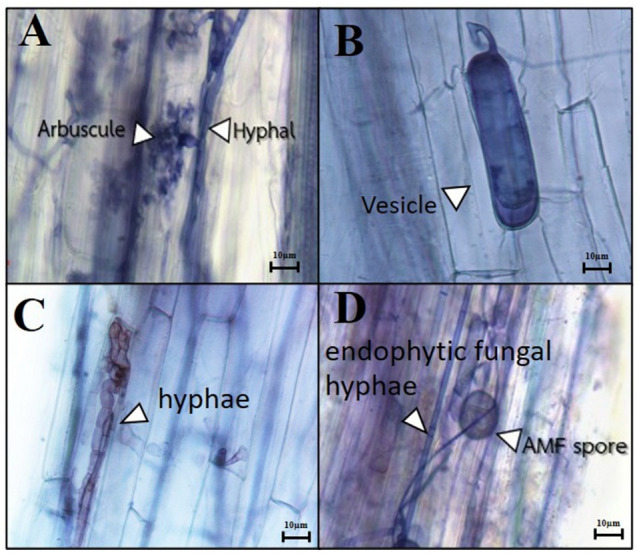
Root colonization of AMF and *Trichoderma*. (**A**) AMF arbuscules (arrow head); (**B**) AMF vesicles (arrow head); (**C**) *Trichoderma* hyphae (arrow head); and (**D**) characteristics of AMF and *Trichoderma* colonization in rice roots.

Mycorrhizal fungal structures, such as hyphae, arbuscules, and vesicles, and *Trichoderma* structures, such as hyphae, were observed (Figure 1). The effects of a single inoculum of AMF (*R. variabilis* KS-02), a single inoculum of *Trichoderma* (*T. zelobreve* PBMP16), and the co-inoculation of AMF and *Trichoderma* on the root colonization and spore number of AMF are shown in Table 5. The highest percentages of AMF colonization in plant roots were found in the treatments with a single inoculum of AMF (T3) and co-inoculation (T5), with significantly higher values than the control treatment (T1). The results also show that the number of AMF spores in soil was only detectable in the treatments involving AMF + *Trichoderma* (T5) and a single inoculum of AMF (T3). This suggests that no other AMF species, apart from *R. variabilis* KS-02, contaminated the soil used in this study. Furthermore, the highest *Trichoderma* root colonization rates of 80.84 and 70.00% were found in the treatments involving *Trichoderma* inoculation (T4 and T5, respectively). A significantly lower rate of *Trichoderma* colonization (below 15%) was observed in the treatments without *Trichoderma* inoculation. These results indicate successful PGPF symbiosis with plants.

### 2.3. The Effects of PGPF on the Phytochemical Properties Under Field Conditions

Table 6 presents the concentrations of functional compounds accumulated in Maled Phai plants following different treatments. The results clearly show that the application of PGPF, either as a single inoculum (T3 and T4) or through co-inoculation (T5), could significantly enhance the content of phenolic compounds compared to the control (T1) and chemical fertilizer treatment (T2). In this regard, Maled Phai plants inoculated with a single inoculum of AMF and those co-inoculated with AMF and *Trichoderma* exhibited the highest total phenolic compound contents of 226.37 and 211.03 mg/L, respectively. Likewise, seeds harvested from both treatments exhibited similar DPPH scavenging activity, with the highest values compared to the other treatments. The application of PGPF alone increased the phenolic compound content and %radical scavenging, even though it had a slightly smaller effect than AMF and co-inoculation. Moreover, AMF inoculation stimulated the accumulation of anthocyanin more than the other treatments. These results suggest that PGPF could better facilitate the accumulation of functional compounds in Maled Phai rice than chemical fertilizer.

The correlation analysis of the AMF spore number, percentage of PGPF colonization, plant growth performance parameters, and concentrations of secondary metabolites in rice seeds are shown in Table 7. The results show that AMF colonization and the AMF spore number had a significant positive effect on the rice grain weight, shoot dry weight, root dry weight, root length, root surface area, root volume, phenolic compound content, anthocyanin concentration, the concentration of nitrogen, and potassium uptake in plants. In contrast, the correlation between the AMF spore number, AMF colonization, Trichoderma colonization, and phosphorus concentration was not significantly different. Moreover, Trichoderma colonization was significantly positively correlated with the tiller number, panicle number, grain weight, shoot dry weight, root dry weight, root surface area, DPPH, and potassium concentration uptake in plants. These results confirm that PGPFs are crucial in increasing the growth and yield of Maled Phai rice.

## 3. Discussion

The overall results indicate that a single inoculum of AMF was the best treatment to increase photosynthesis and the production of functional compounds. This is because AMF can promote the accumulation of various macro- and micronutrients, which help increase photosynthate production, thereby improving biomass production. Furthermore, improved photosynthetic efficiency and nutrient levels, especially P, regulate the synthesis of secondary metabolites [16]. A previous study also showed that micronutrients, such as Zn and Cu, play a role in facilitating the production of secondary metabolites in many types of plants [17]. Our results show that AMF significantly improved root biomass, which is consistent with the findings of Gebremeskel et al. [18]. These findings show that nutrient acquisition from root systems through AMF colonization is an important factor contributing to the overall performance of plants. In addition, the present study showed that a single inoculum of PGPF affected most parameters of plant growth and production and root growth, which is consistent with the observations of Sun et al. [19]. PGPF can modify plant growth through direct action on their hormonal pathways and promote growth based on increased aerial and root biomass, root length, and stem length [20].

Symbiosis between AMF and *Trichoderma* in plants has been reported to enhance plant growth in various types of crops, including rice [21]. It is speculated that *T. zelobreve* PBMP16, a PGPF used in this study, plays a role in enhancing plant performance via the production of plant growth hormones such as cytokinin, auxins, and gibberellins [15]. Such mechanisms were also found in other species of PGPF, as reported by Rodrigues et al. [22]. The results show that a single inoculum and the co-inoculation of AMF and *Trichoderma* had a significant effect, namely through increasing the N and K contents in the rice leaves, making them higher than those in the control plants. Similar results were reported by Chen et al. [23]. In contrast, phosphorus uptake was not significantly affected by any type of treatment, likely because P was directly affected by increased plant or seed yield [24], and higher concentrations of bioactive compounds such as phenolics and anthocyanins had an additional indirect effect [25]. In a study on *Cannabis sativa* [26], an increase in bioactive compounds was correlated with phosphorus acquisition. Similar results were reported by Abdel-Mawgoud et al. [27], who found that the P content in treated soybean plants had a non-significant effect. This suggests that AMF form a network of hyphae that extends beyond the plant’s roots, exploring the soil for nutrients. AMF can produce enzymes that help solubilize nutrients, making them more accessible to the plant [28]. The utilization of PGPF may enhance the absorption of macro- and micronutrients in plants; a process involved in increasing nutrient uptake includes the production of phytochemical-like substances that favor root growth and increase mass flow or roots’ interception of nutrients, and the secretion of substances such as hydrolytic enzymes, which increase nutrient solubilization [29].

Although the application of PGPMs as a replacement for chemical fertilizer to promote plant growth and yield is promising, its utilization in field settings is still challenging. To obtain better plant performance until harvesting, inoculated PGPF should maintain their population density throughout the plant growth period. A few studies indicated that the establishment and persistence of commercial AMF strains were limited when applied in the fields due to competition with well-adapted indigenous communities [30]. This suggests that the selection of native PGPF isolates could lead to more successful results. Considering that many different microbial species coexist in the same field, it is crucial to further study the use of different mixes of PGPF cultures to achieve effective microbial communities to enhance plant growth and yield. In this study, the co-inoculation of two fungal PGPF, namely an AMF species, *R. variabilis* KS-02, and a *Trichoderma* strain, *T. zelobreve* PBMP16, could enhance the biomass and yield of the Maled Phai rice variety. Moreover, these fungal strains also increased the quality of Maled Phai rice seeds by increasing the production of functional compounds, which is a signature trait of this rice variety. This work is the first to provide evidence that a combination of both fungi was effective in improving the growth and productivity of Maled Phai rice grown under field conditions, showing that its performance was even better than that with the use of the chemical fertilizer. Therefore, this finding paves the way for the development of a biofertilizer for the cultivation of rice under field conditions.

## 4. Materials and Methods

### 4.1. Preparation of AMF Inoculum in Greenhouse Condition

The AMF species *Rhizophagus variabilis* KS-02 (Accession No. OQ456401) used in this work was obtained from the Mycotechnology Laboratory, Department of Microbiology, Khon Kaen University, Thailand. Previously, this fungus showed the best results in increasing the grain yield and bioactive compounds of Maled Phai rice in a pot trial [14]. AMF spore multiplications were carried out using a pot culture technique, as described by Boonlue et al. [31]. Briefly, soil (5 kg) was sterilized twice by using an autoclave at 121 °C for 2 h and then placed into 20 cm diameter plastic pots. Maize (*Zea mays* L.) seeds were surface-sterilized through soaking in 10% sodium hypochlorite solution for 30 min prior to sowing in pots. Then, AMF spores in soil inoculants (10 g soil, 40 spores/g) were added to the pots containing maize seeds. Maize was subsequently grown in a greenhouse at 30–35 °C and irrigated with tap water every day. After 90 days, irrigation was stopped and the plants were left to dry out, leading to AMF sporulation. The plants were cut off at a position just above the soil surface. After that, the soil was air-dried and then ground into fine particles (<0.2 mm). The purity of the spores and the total number of spores in the soil were determined using the sucrose centrifugation method [32]. Dried soils containing AMF spores, mycelia, and colonized root fragments were then used as the inoculum.

### 4.2. Preparation of Trichoderma Inoculum

The *Trichoderma* used in this study was *Trichoderma zelobreve* PBMP16 (Accession No. LC738613) and was obtained from the Mycotechnology Laboratory, Department of Microbiology, Khon Kaen University, Thailand. It was used because of its efficiency in enhancing the growth and yield of Maled Phai rice cultivated in pot trials under greenhouse conditions [15]. *T. zelobreve* was cultured on potato dextrose agar (PDA) and incubated at 25 ± 2 °C for 3 days. Subsequently, PDA-containing fungal mycelia were cut using a 0.5 cm diameter cork-borer and then transferred to bottles containing 30 g of steam-sterilized sorghum grains. The bottles were statically incubated at ambient temperature for 14 days or until full colonization occurred [33]. After that, sorghum seeds with full fungal colonization were used as an inoculum.

### 4.3. Experimental Design and Rice Cultivation

The field experiments were set up at the Agronomy Farm, Khon Kaen University, Thailand (16°28′ N, 102°48′ E, 200 m above mean sea level). A Randomized Complete Block Design (RCBD) with four replications was carried out within a plot size of 15 m^2^ (width 3 m × length 5 m), where each plot consisted of 220 plants (11 rows × 20 plants). Plant spacing was 0.25 m between each plot and 0.25 m between each plant within the same row. The study was performed from November 2022 to February 2023. The climate consists of an average temperature of 18–30 °C, partly cloudy with a rate of 63%, 40–45 mm of rainfall, 15–20% of precipitation, 70–80% moisture, 12 hr of sunlight, and a wind speed of 11.2 km/hr. Four treatments were carried out, as follows:

T1: non-inoculated control plants.

T2: plants treated with a full dose of chemical fertilizers (N–P–K of 15–15–15); plants treated with 235 g of the chemical fertilizer in each plot.

T3: plants treated with a single inoculation of AMF (*Rhizophagus variabilis* KS-02).

T4: plants treated with a single inoculation of *Trichoderma* (*Trichoderma zelobreve* PBMP16).

T5: plants treated with the co-inoculation of *R. variabilis* KS-02 and *T. zelobreve* PBMP16.

The chemical and physical properties of soils in the field were determined prior to cultivation. The chemical compositions and physiological properties of the soil were as follows: sandy loam soil with a pH of 6.51, electrical conductivity (EC) of 0.019 dS m^−1^, an organic matter (OM) content of 0.55%, a cation exchange capacity (CEC) of 7.10 (c mol kg^−1^), a total nitrogen (N) content of 290 mg kg^−1^, an available P (method Bray 2) content of 20 mg kg^−1^, an exchangeable K content of 34.64 mg kg^−1^, a calcium (Ca) content of 3171.76 mg kg^−1^, a magnesium (Mg) content of 62.23 mg kg^−1^, and a sodium (Na) content of 339.35 mg kg^−1^.

Black upland rice (*Oryza sativa* subsp. *indica*) or Maled Phai was provided by the group involved in the Rice Project, Faculty of Agriculture, Khon Kaen University, Thailand. Ten rice grains were transferred into a 5 cm deep hole. In the treatments containing AMF (T3 and T5), an AMF inoculum of approximately 1000 spores g^−1^ soil was inoculated per hole. In the treatments using endophytic fungi, 10 sorghum seeds infected with *Trichoderma* were placed beneath rice grains in each hole (T4 and T5). In the case of treatment T2, a chemical fertilizer, N-P-K (235 g per plot), was added after 30 days of transplantation. Plants were irrigated using a mini sprinkler system for 30 min once a day to maintain sufficient humidity. Manual weeding was carried out every 4 weeks after transplanting. Pests and diseases were not controlled until harvest.

### 4.4. Determination of Plant Growth Parameters

At the harvesting stage (120 days after transplantation), 10 plants from each of the four replications (sample number per treatment, 10 × 4 = 40n) were randomly collected to determine the plant growth parameters. The parameters included the plant height, SPAD chlorophyll meter reading (SCMR) (recorded using a chlorophyll meter SPAD-502 plus (Konica Minolta, Shinagawa, Japan), the number of tillers, and the number of panicles.

To quantify the leaf chlorophyll content, fresh leaves were cleaned thoroughly and then ground. After that, 1 g of ground leaves was used for chlorophyll extraction using 80% acetone. The mixture was centrifuged at 4000 rpm for 10 min to retrieve the supernatant. Chlorophyll contents were determined by measuring absorbance at a wavelength of 663 nm (Abs663) and 645 nm (Abs645) using a spectrophotometer (Hitachi High-Tech Science Corporation, Tokyo, Japan). The chlorophyll a, chlorophyll b, and total chlorophyll contents were calculated according to [34] the equations shown below:

Chlorophyll a content = (12.7 × Abs663) − (2.69 × Abs645)

Chlorophyll b content = (22.9 × Abs645) − (4.68 × Abs663)

Total chlorophyll content = (20.2 × Abs645) + (8.02 × Abs663)

To determine plant biomass, the leaves, stems, and roots were dried in an oven at 80 °C for 3 days prior to gravimetric analysis. Then, the grain yield and harvest index (HI) were calculated. The quality of plant roots was determined by measuring the root length, diameter, surface area, volume, specific root length, and root tissue density by scanning the root samples using an Epson scanner V800 PHOTO. The data were analyzed using WINRHIZO Pro2004a software (REGENT Instruments Inc., Quebec, QC, Canada).

The second expanded leaves from the top were analyzed to determine the photosynthesis rate, stomatal conductance, and transpiration rate using an LI-6400XT portable photosynthesis system (LI-COR Bioscience, Lincoln, USA). The water use efficiency (WUE) was calculated by dividing the photosynthetic rate by the transpiration rate.

### 4.5. Nutrient Contents

Shoot samples (stems and leaves) were used to determine the nutrient concentrations (nitrogen (N), phosphorus (P), and potassium (K)). The total N content was extracted from plant tissues using the micro-Kjeldahl method [35]. Then, the N content was analyzed with the colorimetric method using an Auto-Analyzer 3 ((AA3), SEAL Analytical, Germany; Method No. G-253-00 Rev.1 (Multitest MT7/MT8)) at an absorbance of 660 nm. Phosphorus was extracted using the wet oxidation method via mixing with nitric acid and perchloric acid. The total P content was determined using a spectrophotometer at a wavelength of 420 nm and molybdovanadate using the acid persulfate digestion method [36]. Potassium was extracted from plant tissues using the wet oxidation method via mixing with nitric acid and perchloric acid (2:1 *v*/*v*). Then, the K content in the solution was detected using a flame photometer at 768 nm (flame photometer, Model 410, Sherwood, United Kingdom) [37].

### 4.6. Determination of Phytochemical Compounds in Rice Seeds

The phytochemical compounds in rice seeds, including the total anthocyanin concentration (TAC), total phenolic compounds (TPCs), and 1,1-diphenyl-2-picrylhydrazyl radicals (DPPH), were analyzed. These compounds were extracted according to the method described by Kapcum et al. [3] with some modifications. One gram of ground dried seeds was subjected to extraction with 10 mL of methanol. The mixture was shaken for 2 h and then centrifuged at 3000 rpm for 10 min. The mixture was filtered using Whatman No.1 filter paper, and the residues were subjected to re-extraction twice with 5 mL of methanol following the same procedure. Triplicate aliquots of each extract were combined and then stored in the dark at −40 °C until analysis.

To determine the TAC, 50 µL of extract was mixed with 3 mL of 0.025 M of KCl buffer at pH 1.0. Another aliquot of 50 µL of extract was mixed with 0.4 M of sodium acetate buffer at pH 4.5. Each mixture was then left undisturbed at ambient temperature for 20 min prior to absorbance measurement at 520 and 700 nm. The TAC was calculated using the equation. The results were expressed as the amount of cyanidin-3-glucoside equivalent to 100 g of the sample [38]:

Total anthocyanins (mg/100 g) = (∆A × MW × D × (V/G) × 100)/(ϵ × L)

where ∆A is absorbance = (A520 nm − A700 nm) pH 1.0 − (A520 nm − A700 nm) pH 4.5; ϵ is the molar extinction coefficient of Cy-3-G = 29,600 M^−1^ cm^−1^; L is the cell path length of cuvette = 1 cm; MW is the molecular weight of anthocyanins = 449.2 g mol^−1^; D is a dilution factor; V is the final volume (mL); and G is the weight of the sample (g).

To determine the TPCs, 125 µL of extract was mixed with 250 µL of Folin–Ciocalteu’s reagent, followed by the addition of 3 mL of distilled water. The solution was mixed well and then left to stand for 6 min. After that, 2.5 mL of 7% sodium carbonate solution was added. The reaction mixture was left at room temperature for 90 min before measuring absorbance at 760 nm (Hitachi High-Tech Science Corporation, Tokyo, Japan). Gallic acid was used as a calibration standard. The results were expressed as mg of gallic acid equivalent to 100 g of sample [39].

DPPH free radical scavenging activity was determined according to the method described by Leong and Shui [40] with some modifications. A fresh solution of 0.1 mM of DPPH in methanol was prepared. An aliquot of 100 µL of each sample (with appropriate dilution) was mixed with 4.0 mL of 0.1 mM DPPH solution and then left to stand at room temperature for 30 min prior to absorbance measurement at wavelengths of 515 and 517 nm using a spectrophotometer (Hitachi High-Tech Science Corporation, Tokyo, Japan). The percentage of radical scavenging ability was calculated using the following equation:

$$\%\;\mathrm{DPPH}\;\mathrm{scavenging}=\frac{\left(A0-A1\right)}{A0}\times 100$$

where A0 is the absorbance at a 515 nm wavelength and A1 is the absorbance of the sample at a 517 nm wavelength.

### 4.7. Determination of PGPF Colonization in Plant Roots

The percentage of plant root colonization was determined from four plants in each experimental plot. The staining of plant roots was carried out following a method described by Koske and Gemma [41] with some modifications. Fresh roots were washed with tap water and then sieved to remove soil. Roots were decolorized by soaking in 2.5% KOH solution for 10 min at 90 °C. After that, 0.05% lactic acid–glycerol–Trypan blue was used to stain the root samples. The root samples were cut into 0.5–1.0 cm pieces and placed on glass slides to visualize them under a microscope. AMF structures and their root colonization were observed using a light microscope. Observations of the *Trichoderma* present in the roots were carried out in a similar manner to the AMF colonization assessment. *Trichoderma* root colonization was observed in 30 root fragments from different zones of the roots for each treatment. The colonization frequency of PGPF in roots was determined according to the method described by Mehmood et al. [42] using the following equation:

$$\%\;\mathrm{PGPF}\;\mathrm{colonization}\;\mathrm{frequency}=\frac{Total\;number\;of\;root\;colonized}{Total\;number\;of\;roots}\;\times 100$$

### 4.8. Quantification of AMF Spores

Several AMF spores extracted from soil samples were quantified following the method described by Daniels and Skippe [43]. Briefly, 5 g of soil samples was mixed with 30 mL of tap water. The soil suspension was centrifuged at 5000 rpm for 5 min to remove the supernatant. Then, 30 mL of sucrose solution (50% (*w*/*v*)) was mixed with the soil pellet. The mixture was centrifuged at 3000 rpm for 1 min to detach AMF spores from the soil. The supernatant was filtered through a Whatman No.1 filter paper and then placed on a Petri dish to count spores under a stereomicroscope (Nikon SMZ745T).

### 4.9. Statistical Analysis

The data were analyzed using a one-way analysis of variance (ANOVA). The data were tested for normality and homogeneity of variances. The least significant difference (LSD) test was applied to test for significant differences among the means of different treatments at a *p*-value < 0.05. The correlation between parameters was calculated using Pearson’s correlation coefficient and evaluated at a *p*-value < 0.05. All statistical analyses were performed using Statistix 10 software.

## 5. Conclusions

In summary, the application of PGPF inoculation to promote the growth, yield, and phytochemical compounds of Maled Phai rice demonstrated significant advantages over the use of chemical fertilizers and proved effective under field conditions. These findings pave the way for the further development of AMF and *Trichoderma* inocula in the industrial production of biofertilizers as a replacement for synthetic fertilizers. Due to the environmentally friendly nature of AMF and endophytic fungi, they have positive effects on plant performance, yield, and phytochemical concentrations. This approach supports the establishment of more sustainable production systems, offering farmers an environmentally friendly and sustainable alternative for rice cultivation.

## Figures and Tables

**Table 1:** Effects of plant growth-promoting fungi on the growth and production of Maled Phai at the harvesting stage.

Treatments	Plant Height (cm)	Number of Tillers	Shoot Dry Weight (g)	Number of Panicles	Grain Weight (kg/ha)	Harvest Index (HI)
T1	88.64 ^c^	9.93 ^d^	21.52 ^d^	7.00 ^c^	1310.70 ^c^	0.33 ^a^
T2	96.29 ^b^	13.13 ^c^	29.75 ^c^	9.00 ^b^	2416.70 ^b^	0.38 ^a^
T3	98.84 ^ab^	16.08 ^b^	44.45 ^b^	11.00 ^a^	3420.40 ^a^	0.32 ^a^
T4	100.65 ^a^	19.18 ^a^	54.56 ^a^	12.00 ^a^	4120.40 ^a^	0.33 ^a^
T5	96.53 ^b^	17.33 ^ab^	53.11 ^a^	11.00 ^a^	4034.40 ^a^	0.30 ^a^
% CV	2.42	12.17	10.92	8.34	20.13	16.46
F-test	**	**	**	**	**	ns

Numbers followed by the same letter in each column were not significantly different according to the LSD test. ns, non-significant difference; ** significant difference at *p* ≤ 0.01. (T1: control; T2: chemical fertilizer; T3: AMF; T4: *Trichoderma*; and T5: AMF + *Trichoderma*).

**Table 2:** Effects of plant growth-promoting fungi on chlorophyll content and photosynthesis-related characteristics of Maled Phai at the harvesting stage.

Treatments	Pn (µmol CO_2_ m^−2^ s^−1^)	gs (H_2_O m^−2^ s^−1^)	Tr (mmol H_2_O m^−2^ s^−1^)	WUE (µmol CO_2_/H_2_O m^−2^ s^−1^)	SPAD Values	Chlorophyll Content (mg/L)
T1	13.10 ^b^	0.18 ^a^	2.67 ^a^	4.91 ^b^	28.40 ^b^	31.27 ^c^
T2	13.85 ^b^	0.17 ^a^	2.87 ^a^	4.81 ^b^	36.03 ^a^	39.63 ^c^
T3	17.33 ^a^	0.16 ^a^	2.62 ^a^	6.62 ^a^	36.20 ^a^	66.54 ^a^
T4	16.90 ^a^	0.14 ^a^	2.44 ^a^	7.00 ^a^	36.32 ^a^	57.02 ^b^
T5	16.73 ^a^	0.16 ^a^	2.60 ^a^	6.52 ^a^	37.20 ^a^	49.91 ^b^
% CV	9.39	20.4	11.29	11.65	3.87	11.29
F-test	**	ns	ns	**	**	**

Numbers followed by the same letter in each column were not significantly different according to the LSD test. ns, non-significant difference; ** significant difference at *p* ≤ 0.01. (T1: control; T2: chemical fertilizer; T3: AMF; T4: *Trichoderma*; and T5: AMF + *Trichoderma*).

**Table 3:** Effects of plant growth promoting-fungi on root growth of Maled Phai at the harvesting stage.

Treatments	Root dry Weight (g)	Length (cm)	Surf Area (cm^2^)	Avg Diam (mm)	Volume (cm^3^)	Specific Root Length (m/g)	Root Tissue Density (g/cm^3^)
T1	2.80 ^b^	662.90 ^c^	138.07 ^b^	1.10 ^bc^	1.95 ^c^	2.41 ^b^	0.45 ^a^
T2	2.80 ^b^	917.20 ^b^	129.09 ^b^	0.90 ^d^	1.50 ^d^	3.34 ^a^	0.47 ^a^
T3	4.53 ^a^	1115.70 ^a^	175.19 ^a^	1.17 ^a^	2.58 ^a^	2.48 ^b^	0.38 ^a^
T4	3.97 ^a^	950.20 ^b^	165.31 ^a^	1.14 ^ab^	2.39 ^ab^	2.40 ^b^	0.41 ^a^
T5	4.31 ^a^	1154.80 ^a^	166.05 ^a^	1.05 ^c^	2.24 ^bc^	2.71 ^b^	0.44 ^a^
%CV	11.77	5.39	10.43	3.96	10.12	12.96	14.89
F-test	**	**	**	**	**	*	ns

Numbers followed by the same letter in each column were not significantly different according to the LSD test. ns, non-significant difference; ** significant difference at *p* ≤ 0.01; * significant difference at *p* ≤ 0.05. (T1: control; T2: chemical fertilizer; T3: AMF; T4: *Trichoderma*; and T5: AMF + *Trichoderma*).

**Table 4:** Effects of plant growth-promoting fungi on plant nutrient uptake of Maled Phai at the harvesting stage.

Treatments	Total Nitrogen (g/kg)	Total Phosphorus (g/kg)	Total Potassium (g/kg)
T1	4.45 ^c^	1.78 ^a^	16.15 ^b^
T2	5.50 ^ab^	2.05 ^a^	18.21 ^ab^
T3	5.74 ^ab^	1.98 ^a^	19.70 ^a^
T4	5.28 ^b^	1.89 ^a^	19.96 ^a^
T5	6.01 ^a^	2.16 ^a^	20.23 ^a^
% CV	8.41	12.51	7.52
F-test	**	ns	**

Numbers followed by the same letter in each column were not significantly different according to the LSD test. ns, non-significant difference; ** significant difference at *p* ≤ 0.01. (T1: control; T2: chemical fertilizer; T3: AMF; T4: *Trichoderma*; and T5: AMF + *Trichoderma*).

**Table 5:** Total AMF spores and percentage of plant growth-promoting fungi root colonization at the harvesting stage of Maled Phai.

Treatments	Number of AMF Spores (Spore Soil^−1^)	AMF Colonization (%)	*Trichoderma* Colonization (%)
T1	0.00 ^c^	2.67 ^b^	5.84 ^b^
T2	0.00 ^c^	5.54 ^b^	7.50 ^b^
T3	2.39 ^b^	32.91 ^a^	13.33 ^b^
T4	0.10 ^c^	5.54 ^b^	80.84 ^a^
T5	2.70 ^a^	32.93 ^a^	70.00 ^a^
% CV	6.91	15.9	27.55
F-test	**	**	**

Numbers followed by the same letter in each column were not significantly different according to the LSD test. ns, non-significant difference; ** significant difference at *p* ≤ 0.01. (T1: control; T2: chemical fertilizer; T3: AMF; T4: *Trichoderma*; and T5: AMF + *Trichoderma*).

**Table 6:** Concentration of anthocyanin, total phenolic compounds, and the antioxidant capacity of Maled Phai seeds.

Treatments	Phenolic Compound (mg/L)	Anthocyanin (mg. Cy-3-G eq./100 g)	Antioxidant (% DPPH Scavenging)
T1	166.41 ^c^	56.84 ^b^	91.23 ^c^
T2	164.02 ^c^	53.17 ^b^	91.92 ^bc^
T3	226.37 ^a^	72.97 ^a^	94.03 ^ab^
T4	190.51 ^b^	59.43 ^b^	92.61 ^abc^
T5	211.03 ^a^	57.67 ^b^	94.38 ^a^
% CV	5.34	8.86	1.52
F-test	**	**	*

Numbers followed by the same letter in each column were not significantly different according to the LSD test. ns, non-significant difference; ** significant difference at *p* ≤ 0.01; * significant difference at *p* ≤ 0.05. (T1: control; T2: chemical fertilizer; T3: AMF; T4: *Trichoderma*; and T5: AMF + *Trichoderma*).

**Table 7:** Correlation between AMF and Trichoderma inoculation and plant growth parameters of rice at the harvest stage.

Correlation	Till	Pani	Grain	Shoot	Root	Length	Surf	Diam	Vol	SRL	RTD	Pheno	Antho	DPPH	N	P	K	Spore	AMF
Panicle number	0.90 **																		
Grain weight	0.79 **	0.83 **																	
Shoot dry weight	0.83 **	0.80 **	0.91 **																
Root dry weight	0.61 **	0.67 **	0.63 **	0.75 **															
Root length	0.53 *	0.56 **	0.72 **	0.76 **	0.76 **														
Root surface area	0.49 *	0.42 ns	0.66 **	0.70 **	0.67 **	0.61 **													
Root diameter	0.24 ns	0.22 ns	0.32 ns	0.34 ns	0.50 *	0.10 ns	0.52 *												
Root volume	0.47 *	0.42 ns	0.60 **	0.64 **	0.70 **	0.47 *	0.93 **	0.79 **											
SRL	−0.23 ns	−0.23 ns	−0.05 ns	−0.19 ns	−0.53 *	0.12 ns	−0.34 ns	−0.67 **	−0.55 *										
RTD	−0.10 ns	−0.03 ns	−0.39 ns	−0.27 ns	−0.09 ns	−0.30 ns	−0.37 ns	−0.59 **	−0.47 *	−0.19 ns									
Phenolic compound	0.46 *	0.42 ns	0.56 **	0.62 **	0.81 **	0.74 **	0.82 **	0.47 *	0.77 **	−0.33 ns	−0.30 ns								
Anthocya-nin	0.27 ns	0.32 ns	0.39 ns	0.26 ns	0.46 *	0.35 ns	0.48 *	0.66 **	0.61 **	−0.24 ns	−0.60 **	0.66 **							
DPPH	0.43 ns	0.34 ns	0.31 ns	0.34 ns	−0.10 ns	0.11 ns	−0.08 ns	−0.44 ns	−0.25 ns	0.31 ns	0.24 ns	−0.28 ns	−0.49 *						
N	0.36 ns	0.43 ns	0.51 *	0.46 *	0.49 *	0.64 **	0.32 ns	−0.08 ns	0.20 ns	0.13 ns	−0.03 ns	0.62 **	0.30 ns	0.08 ns					
P	0.21 ns	0.21 ns	0.30 ns	0.24 ns	0.13 ns	0.35 ns	0.17 ns	−0.20 ns	0.03 ns	0.21 ns	0.00 ns	0.30 ns	−0.04 ns	0.13 ns	0.43 ns				
K	0.60 **	0.48 *	0.5 6 **	0.67 **	0.57 **	0.65 **	0.54 *	0.17 ns	0.45 *	−0.05 ns	−0.20 ns	0.60 **	0.16 ns	0.42 ns	0.58 **	0.39 ns			
AMF spore number	0.35 ns	0.39 ns	0.48 *	0.51 *	0.74 **	0.78 **	0.57 **	0.29 ns	0.52 *	−0.14 ns	−0.23 ns	0.82 **	0.50 *	−0.33 ns	0.60 **	0.32 ns	0.48 *		
AMF colonization	0.34 ns	0.43 ns	0.51 *	0.51 *	0.74 **	0.80 **	0.55 *	0.29 ns	0.51 *	−0.12 ns	−0.25 ns	0.81 **	0.53 *	−0.34 ns	0.60 **	0.35 ns	0.45 *	0.98 **	
Trichoderma colonization	0.60 **	0.53 *	0.72 **	0.80 **	0.45 *	0.43 ns	0.47 *	0.26 ns	0.42 ns	−0.19 ns	−0.17 ns	0.29 ns	−0.11 ns	0.47 *	0.25 ns	0.06 ns	0.53 *	0.21 ns	0.18 ns

ns, non-significant difference; ** significant difference at *p* ≤ 0.01; * significant difference at *p* ≤ 0.05.

## Data Availability

The datasets obtained and analyzed in the current study are available from the corresponding author upon reasonable request.

## Supplementary Materials

The following are available online at https://www.mdpi.com/article/10.3390/plants14121839/s1, Figure S1: Rice growing on the field. (A), set up a sprinkler watering system and (B), weed control at 15 days after cultivation. Figure S2: Rice growing on the field. (A), after 30 days of rice cultivation and (B), after 60 days of rice cultivation. Figure S3: Rice grows on the field. (A), after 90 days of rice cultivation and (B), install netting to keep out birds. Figure S4: Rice harvest. (A), the yellow rice seeds after 120 days of cultivation and (B), the rice seeds are dried in the sun for 7 days.
