# Supplementary material for: The Potential of Plant Growth-Promoting Fungi Enhances the Growth, Yield, and Phytochemical Compounds of Oryza sativa L. (Maled Phai Cultivar) Under Field Conditions
Source: Plants (Basel). 2025 Jun 15;14(12):1839. doi: 10.3390/plants14121839 (PMC12196539; doi:10.3390/plants14121839)
Supplement: Supplementary file 1 [file plants-14-01839-s001.zip › plants-3646604-supplementary.pdf]

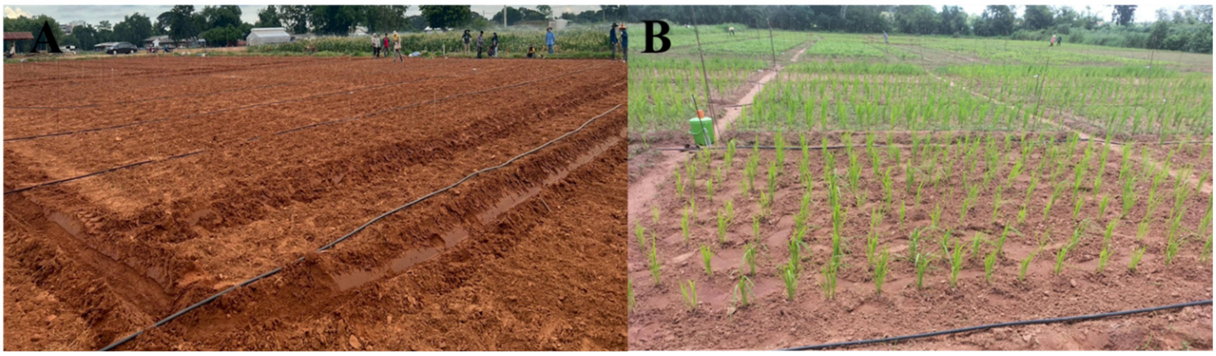

Figure 1. Rice growing on the field. A), set up a sprinkler watering system and B), weed control at 15 days after cultivation.

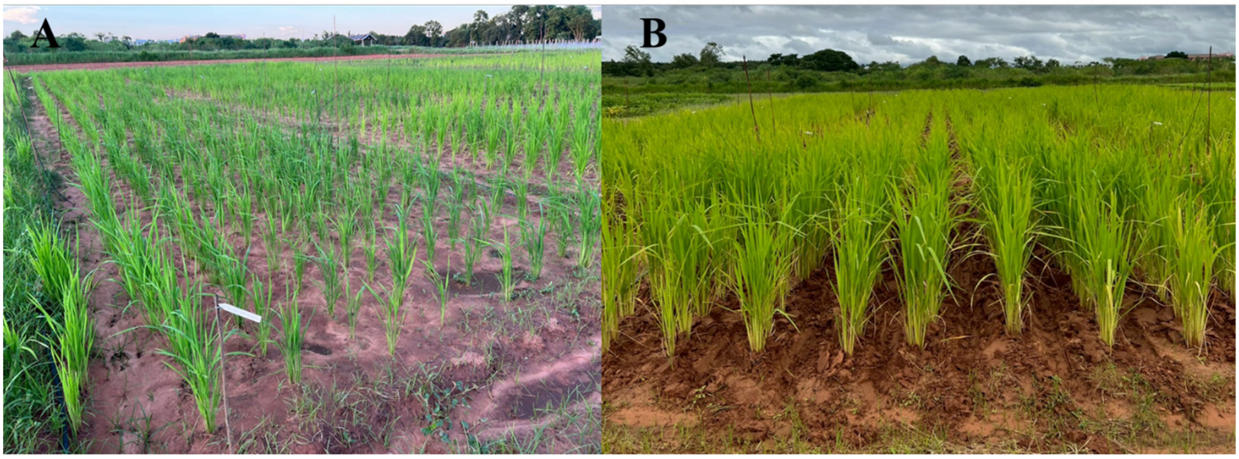

Figure 2. Rice growing on the field. A), after 30 days of rice cultivation and B), after 60 days of rice cultivation.

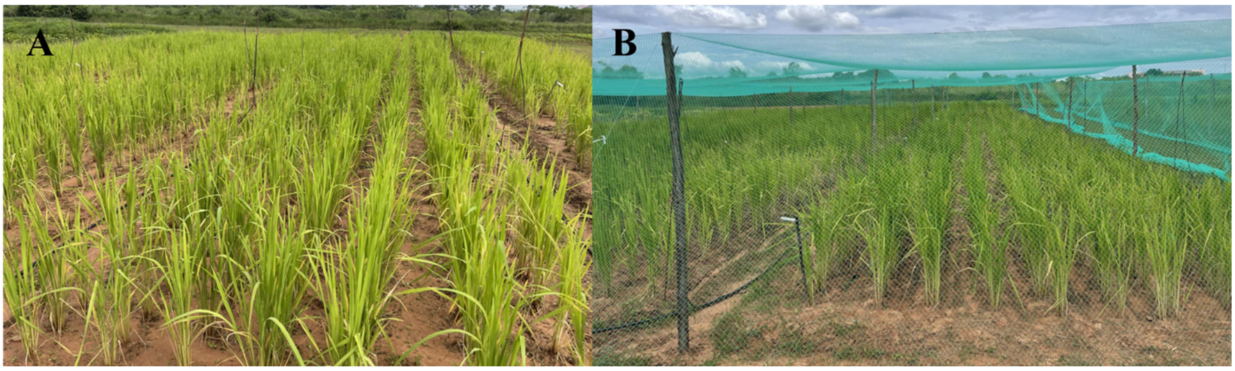

Figure 3. Rice grows on the field. A), after 90 days of rice cultivation and B), install netting to keep out birds.

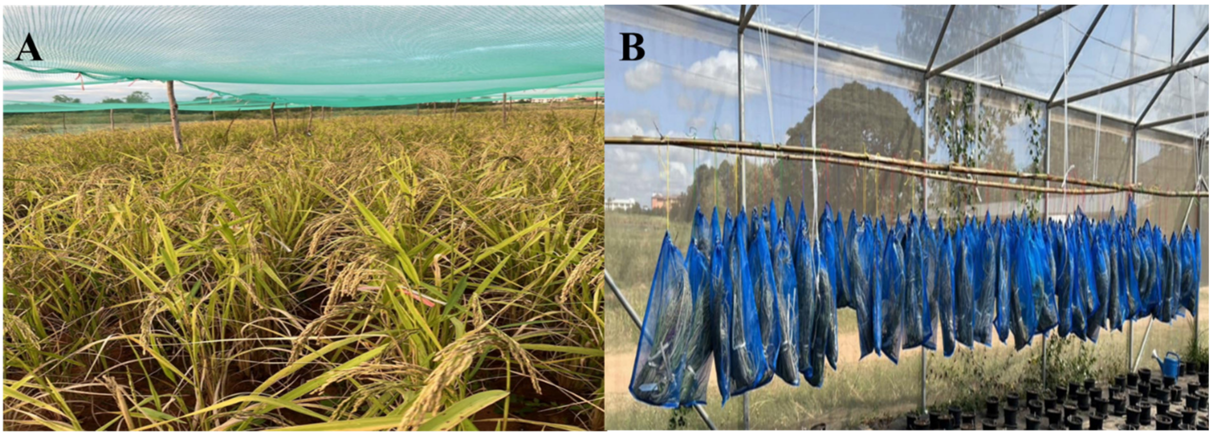

Figure 4. Rice harvest. A), the yellow rice seeds after 120 days of cultivation and B), the rice seeds are dried in the sun for 7 days.
